# Learned Manipulation at Unconstrained Contacts Does Not Transfer across Hands

**DOI:** 10.1371/journal.pone.0108222

**Published:** 2014-09-18

**Authors:** Qiushi Fu, Jason Y. Choi, Andrew M. Gordon, Mark Jesunathadas, Marco Santello

**Affiliations:** 1 School of Biological and Health Systems Engineering, Arizona State University, Tempe, Arizona, United States of America; 2 Department of Biobehavioral Sciences, Teachers College, Columbia University, New York, New York, United States of America; University of Reading, United Kingdom

## Abstract

Recent studies about sensorimotor control of the human hand have focused on how dexterous manipulation is learned and generalized. Here we address this question by testing the extent to which learned manipulation can be transferred when the contralateral hand is used and/or object orientation is reversed. We asked subjects to use a precision grip to lift a grip device with an asymmetrical mass distribution while minimizing object roll during lifting by generating a compensatory torque. Subjects were allowed to grasp anywhere on the object’s vertical surfaces, and were therefore able to modulate both digit positions and forces. After every block of eight trials performed in one manipulation context (i.e., using the right hand and at a given object orientation), subjects had to lift the same object in the second context for one trial (transfer trial). Context changes were made by asking subjects to switch the hand used to lift the object and/or rotate the object 180° about a vertical axis. Therefore, three transfer conditions, hand switch (HS), object rotation (OR), and both hand switch and object rotation (HS+OR), were tested and compared with hand matched control groups who did not experience context changes. We found that subjects in all transfer conditions adapted digit positions across multiple transfer trials similar to the learning of control groups, regardless of different changes of contexts. Moreover, subjects in both HS and HS+OR group also adapted digit forces similar to the control group, suggesting independent learning of the left hand. In contrast, the OR group showed significant negative transfer of the compensatory torque due to an inability to adapt digit forces. Our results indicate that internal representations of dexterous manipulation tasks may be primarily built through the hand used for learning and cannot be transferred across hands.

## Introduction

The ability to perform dexterous manipulation relies on building sensorimotor memories of previous hand-object interactions for anticipatory control of finger forces as well as processing ongoing sensory feedback [1]–[4] (for review see [5]). The nature of the internal representations of manipulation tasks allowing for anticipatory control has been studied extensively by testing subjects’ ability to transfer learned manipulations [6]. For instance, when we learn to grasp and lift a container with unknown contents, we need to adapt our digit positions and forces to balance it. What would happen if, after learning the manipulation task, we subsequently lift the same object in a new orientation and/or with the contralateral hand? A useful experimental approach to address this question is to use learning transfer paradigms in which subjects are tested on whether the manipulation learned in one context may positively or negatively affect the performance of manipulation in a different context. It has been shown that the extent to which learning transfer can occur is sensitive to the type of manipulation tasks. Specifically, if the task requires subjects to uniformly scale fingertip forces (i.e. thumb and index finger forces have to be shared equally) to object properties such as object weight or texture, subjects are able to transfer digit forces to the contralateral hand [1], [7]. However, if subjects learn non-uniform fingertip force distributions as required by the tasks, subjects are unable to transfer asymmetrical force sharing following object rotation or switching the hand used to lift the object [8]–[13].

One major limitation of the above studies is that they constrain contact at predetermined locations on the object such that non-uniform sharing of finger forces was the only solution. Several recent studies have shown that when such digit placement constraints are removed, subjects actively modulate contact points as a function of object properties such as mass distribution [14] or shape [15], [16], as well as planned manipulation [17], [18]. Most importantly, it has been shown that digit placement and forces are not independent and that their trial-to-trial covariation suggests the existence of high-level representations of learned manipulation task, i.e., the net torque that has to be generated for any combination of digit force and position [19], [20]. Therefore, the question arises as to whether the above-described failure of transferring learned digit forces can be extended to unconstrained manipulation in which digit positions and forces have to be learned together to perform a given manipulation. This question was partially addressed by Zhang et al. [21] who asked subjects to lift an object with the same hand after 8 lifts following a 180° object rotation about its vertical axis. To successfully manipulate the object, subjects had to learn to exert a compensatory moment at object lift onset, in either clockwise (CW) or counter clockwise (CCW) directions, to counter the external torque caused by a hidden mass added at one side of the visually symmetrical object (inverted T-shape). This study revealed that subjects failed to transfer learned compensatory moment after object rotation even with removal of digit placement constraints. Additionally, when subjects were asked to rotate the object after every 8 trials, they gradually improved in their ability to perform the manipulation across subsequent post-rotation trials. This was accomplished primarily by modulation of digit position, and to a lesser extent by modulation of fingertip forces. However, it remains unknown whether a learned manipulation can transfer across hands when the object does not constrain digit placement, and whether learned digit forces and placement transfer to a similar extent across hands.

In this paper we address subjects’ ability to transfer learned manipulation to a second task context performed on the same object following switching the hand used to lift the object as well as object rotation. Specifically, by using an unconstrained object manipulation task similar to [21], we define “learned manipulation” as the ability to combine digit position and force to generate the torque required to prevent object roll, i.e., task performance. We also define “transfer” of learned manipulation as the ability to generate the target torque following a change in manipulation context.

To introduce our hypotheses about learning transfer, we first discuss the change of manipulation context. It has been speculated that sensorimotor learning might occur in different coordinate frames: extrinsic and/or intrinsic frames (R_E_ and R_I_, respectively) [22]. In our task, subjects could learn the object mass distribution in an extrinsic frame (i.e., object torque generated by the hidden mass), or learn the torque produced by the hand (i.e., supination and pronation with respect to the hand/arm muscles). When an object is rotated 180° (OR) after subjects had experienced lifting it, the subsequent manipulation context changes in both R_E_ and R_I_, as subjects need to reverse the torque in R_I_ due to reversal of the object dynamics in R_E_ during subsequent lifts. When subsequent lifts involve using the contralateral hand, i.e., a hand switch (HS), the object dynamics remains unchanged in R_E_ during subsequent lifts, but subjects need to reverse the torque in R_I_ due to the fact that the hands are mirror images of each other (e.g., the target CW torque requires supination of the right hand but pronation of the left hand). When subjects perform subsequent lifts involving both a hand switch and object rotation (HS+OR), the object dynamics reverses in R_E_, but the torque remains unchanged in R_I_. It has been shown that, on the first trial after a change of manipulation context, subjects exhibit a large negative transfer in OR condition, but zero transfer in HS and HS+OR conditions [12]. However, this result differs from findings reported by studies of reaching movements using force fields [22]. Specifically, after switching arm, it was found that positive transfer occurred when the direction of the force field remained the same (similar to HS condition), whereas negative transfer occurred when the direction of the force field reversed (similar to the HS+OR condition). However, this conflicting result might be due to the difference in how learning transfer was assessed. Specifically, manipulation tasks usually only measure the initial bias on the first trial after a change of context [12], [21] since learning occurs within 1 to 2 trials (e.g., [2], [4], [9], [19]). In contrast, studies of reaching tasks measure the rate of learning across multiple trials, which is much slower than learning rates in manipulation tasks. For reaching tasks, the first trial after a context switch does not provide much information about the upcoming task dynamics due to lack of contextual cues. To better evaluate learning transfer of manipulation, our experimental design features a novel trial sequence in which only one trial of the new (second) context (i.e., the one used to assess learning transfer) was tested after each set of initial trials. This allowed us to systematically assess the adaptation occurring across multiple transfer trials. We hypothesized that (H1) positive and negative transfer would occur across multiple transfer trials in the HS and HS+OR conditions, respectively, although the first transfer trial would result in zero transfer. Additionally, we hypothesized that (H2) the OR condition would show negative transfer across all transfer trials.

## Methods

### Subjects

Sixty (21 males, 39 females; age range: 18–39 yrs.) self-reported right-handed subjects participated in the experiment. All subjects were naïve to the experimental procedures and reported that they were without any neurological or orthopedic disorders. Written informed consent was obtained from subjects prior to testing in accordance with the Declaration of Helsinki. The procedures were approved by the Office of Research Integrity and Assurance at Arizona State University.

### Experimental set-up

#### Apparatus

**Figure 1:**
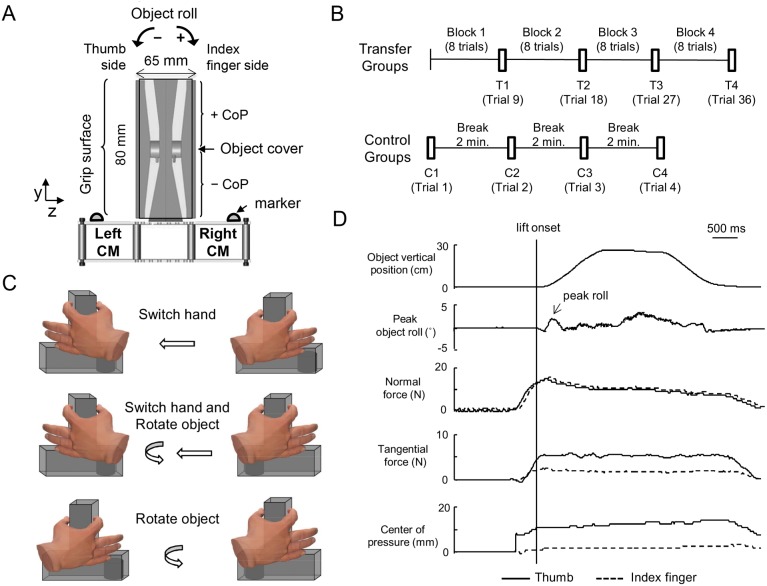
Experimental setup and procedures. Panel A shows the custom-built inverted-T grip device used to measure forces and centers of pressure of the thumb and index finger. Note that ‘thumb side’ and ‘index finger side’ denote a grasp performed with the right hand. Two light-emitting diode markers were mounted on the base of the device to track object kinematics (vertical position and roll in the *x-y* plane). A 400 g mass was inserted in either the left or right compartment to change the center of mass (CM) of the object to create an external moment. A cover was taped on the front and back of the grip device to block view of force/torque sensors. Panel B shows the trial sequence for the transfer groups (upper) and the control groups (lower). Panel C shows three learning transfer conditions with “switch hand” and/or “object rotation”. The example shown denotes a subset of the experimental conditions, i.e., one CM condition per transfer action. Panel D shows data from one representative subject (S9) and the experimental variables: object vertical position, peak object roll, normal and tangential forces of thumb and index finger, and center of pressure for each digit. The sign convention for the digit center of pressure is shown in panel A. Note that the data are from the last trial of Block 1 performed with a left CM object lifted by the right hand. At this stage of the trial sequence, this subject learned to generate a compensatory moment to minimize object roll (<3°). Note that this subject exerted a larger tangential force with the thumb (the added mass was on the thumb side) and the thumb center of pressure was higher than the index finger.

A custom-built inverted T-shaped grip device was used to measure 3-dimensional forces and torques of the thumb and index finger (Figure 1A). Parallel vertical bars covered with 100-grit sandpaper were mounted on each side of the device (length: 8 cm, depth: 2.3 cm; distance between graspable surfaces: 6.5 cm). One 6-axis force/torque transducer was placed perpendicular to each vertical bar to measure fingertip placement (center of pressure, CoP) and forces (normal and tangential forces) (ATI Nano-25 SI-125-3, ATI Industrial Automation, Garner, NC; force range: 125, 125, and 500 N for *x*-, *y*- and *z*-axes, respectively; force resolution: 0.06 N; torque range: 3000 Nmm; torque resolution: 0.378 Nmm). The transducers were mounted collinear with each other on opposite sides of the grip device (Figure 1A). Object center of mass (CM) was changed by inserting a 400 g mass in one of three compartments (left, center, or right) of the object base. The total weight of the object (device plus added mass) was 796 g. Adding the mass to the left and right compartment resulted in a torque of −255 and 255 Nmm, respectively.

Object position and orientation were measured using an active marker 3D motion capture system with eight cameras (frame rate: 480 Hz, spatial resolution: 0.1 mm; Phase Space Inc., San Leandro, CA). Light-emitting diode markers were placed on the top of the left and right compartments of the base (Figure 1A). Force and torque data were acquired with a 12-bit A/D converter (PCI-6225; National Instruments, Austin, TX) and digitized at 1 kHz. Collection of force and object kinematic data was temporally synchronized for each trial using custom designed software (LabView, National Instruments, Austin, TX). After each experimental session, data were stored on a computer for offline processing.

### Experimental procedures

Subjects were instructed to (1) stand in front of the grip device with either left or right shoulder aligned with the grip device, (2) have the corresponding hand rest flat on the table at ∼20 cm from the object while the other hand being relaxed off the table, (3) grasp the object using only the distal pads of the thumb and index finger, (4) for each trial, on a verbal ‘GO’ signal, reach, grasp, and lift the object ∼10 cm at a natural speed, (5) minimize object roll during the lift, (6) hold the object for ∼2 seconds, and (6) replace it on the table. During this process, subjects were constantly reminded to minimize object roll during each lift, as well as to extend the remaining 3 fingers to prevent them from touching the object. Compliance with this requirement was visually verified by one of the experimenters.

Prior to the start of the experiment, subjects were allowed to lift the object two or three times with each hand with the additional 400 g mass in the center location of the base to familiarize themselves with the task, the object’s weight, and frictional properties. After these practice trials, subjects were informed that the mass would be shifted to the left or right for the entire duration of the experiment. Mass location was blocked from view throughout the experiment to prevent giving subjects visual cues to anticipate the direction of the object dynamics on each trial, such that subjects had to learn the correct manipulation through consecutive lifts [23]. Note that correct performance of manipulation required subjects to learn anticipatory control of digit forces and placement to compensate the external moment at object lift onset (see [19], [21] for details). Briefly, subjects had to exert a compensatory moment to counteract the external moment caused by the added mass. Because of reaction time delays, the present manipulation task required subjects to exert a compensatory moment at object lift onset. Note that up to object lift onset, subjects could not sense object CM location.

There were three transfer conditions: “object rotation”, “hand switch”, and “hand switch and object rotation”. Twelve subjects were randomly assigned to each condition (referred to as *transfer groups*). For all transfer groups, the trial sequence consisted of four blocks of eight consecutive trials (“blocked” task trials) and four single “transfer” trials. Each transfer trial was conducted after one block of task trials (Figure 1B). All transfer groups started with their right hand in Block 1, and the object CM condition for the blocked task (right or left CM; R_CM_ or L_CM_, respectively) was counter-balanced across all groups. The difference between transfer groups (HS, OR, and HS+OR) was whether the subjects were asked to rotate the object and/or switch hand (Figure 1C) between blocked task trials and transfer trials.

For the HS group, subjects had to perform the transfer trials with their left hand. Before and after each transfer trial, they were instructed to translate the object to a marked area on the table that was aligned with the contralateral shoulder. The object CM remained unchanged with respect to the extrinsic coordinate frame. For the HS+OR group, subjects also had to perform the transfer trials with their left hand. Before and after each transfer trial, subjects first performed the same object translations as those in HS group, and then they had to also rotate the object 180° about the object’s vertical axis such that the object CM was opposite relative to the trials performed before object rotation. For the OR group, subjects did not use their left hand. They were only required to rotate the object 180° before and after each transfer trial. Note that trials in all blocked tasks had the same context for each subject, whereas transfer trials were characterized by a different context that depended on the action performed before the transfer trial. It should be emphasized that these pre-/post-transfer movements were used to provide strong cues about changes in manipulation context and they are consistent with protocols used by other learning transfer studies of manipulation [9], [12], [21].

Additionally, twenty-four subjects were evenly assigned to a left-hand control group and a right-hand control group. They performed 4 trials with same CM location using their corresponding hand such that their delayed learning trials (C1–C4) could be compared with the transfer learning trials (T1–T4) from the transfer groups (Figure 1B). Note that the break time inserted after each trial (∼2 minutes) in the control groups was equal to the time it took subjects in the transfer groups to perform a block of eight trials. As the control groups experienced no change in manipulation context, their performance on four trials could be compared with the four transfer trials of each transfer group. This allowed us to isolate the effect of blocked trials on transfer trials. Note that these control groups were not used in our previous study where subjects performed blocks of consecutive experimental and transfer trials [21].

### Data processing

Custom written software (Matlab 2013b, The Mathworks Inc., Natick, MA; Microsoft Excel 2010; IBM Statistics SPSS 21) was used for data processing. The aim of the current study was to investigate subjects’ ability to transfer object manipulation learned with one hand and one object orientation to the contralateral hand and/or to the opposite object orientation, i.e., transfer of compensatory moment (see above). If, following switching hand and/or object rotation, subjects could transfer compensatory moment to the new context, object roll minimization learned through a block of consecutive lifts would also transfer. Therefore, the primary variables of interest were *compensatory moment at object lift onset* (when the vertical position of the object crossed a threshold of 0.5 mm for longer than 400 ms) and *peak object roll* (Figure 1D). We also analyzed digit placement and forces to examine how subject performed the tasks through coordination of digits. The analyses focused on the following variables:

1. *Digit forces at object lift onset*: normal (grip) force and digit tangential (load) (F*n* and F*tan*, respectively) exerted by thumb and index finger in the *z*- and *y*-axis of the object, respectively (Figure 1A; Figure 1D).
2. *Digit center of pressure at object lift onset*: the vertical (*y*) coordinate of the point of resultant digit force application relative to the origin of the force/torque transducer (center of pressure, CoP, see [21] for details). The average error of CoP estimation was less than 2 mm. Digit CoP of each digit was defined negative or positive relative to digit positions below or above the origin of the force/torque transducer, respectively (Figure 1A; Figure 1D).
3. *Compensatory moment at object lift onset* (M_com_): the above variables were used to compute compensatory moment as the combination of digit forces and positions [21]. Positive and negative values denote the M_com_ in clockwise and counter clockwise directions with respect to subjects’ body, respectively (Figure 1A).
4. *Digit load force and digit placement strategies*: in our previous studies, we have shown that subjects learned to generate the compensatory moment by modulation of digit load forces and positions [19]. Here, to simplify data analysis, we define digit relative positions ΔCoP as the CoP of the digit on the side of the CM location minus the CoP of the other digit, and digit load force difference ΔF*tan* as the F*tan* exerted by the digit on the side of the CM location minus the F*tan* exerted by the other digit. This definition avoids using sign conventions associated with left/right CM locations as well as the mirrored relationship of thumb and index across right and left hand. For instance, when using the right hand to lift a right CM object, the index finger is on the side of the CM location and a positive ΔCoP would indicate that the index fingertip is positioned higher than thumb tip. In contrast, if the left hand is used to lift a right CM object, a positive ΔCoP indicates that the thumb tip is higher than the index fingertip because the thumb of the left hand is on the side of the CM location. We also define digit grip force F*n* as the mean normal force averaged across thumb and index finger. In this manuscript, we will refer to the ΔCoP as “digit positions”, and both ΔF*tan* and F*n* as “digit forces”. Note that as subjects were not constrained to grasp the object at pre-determined locations on the object, there are theoretically infinite possible combinations of digit placement and forces that would still attain the same task goal (M_com_; [19]).
5. *Peak object roll*: the angular deviation of the object from the vertical on the *y-z* plane during lift. Positive and negative values denote the roll in clockwise and counter clockwise direction, respectively (Figure 1A). Peak object roll was identified to be the initial maximum roll of the object within ∼250 ms of object lift onset. A custom software algorithm was written to determine peak object roll and the lift-off event was visually verified by one of the investigators for each trial. Peak object roll was used to quantify the behavioral consequences of anticipatory control of compensatory torque.

### Statistical analysis

#### Trial-to-trial learning of M_com_ and peak object roll on Block 1

To evaluate subjects’ ability to learn the object manipulation task, we fitted the M_com_ and peak object roll of 8 trials in the first block for all conditions with an exponential decay model *y = ae^−bx^+c* using the Levenberg-Marquardt algorithm (Flanagan et al., 2003). The half-life of this model was computed to quantify rate of blocked learning. We also applied regression analysis to compensatory moment and peak object roll.

#### Learning, transfer, and post-transfer comparisons

To avoid complication caused by using different signs of M_com_ for each CM condition, for statistical analysis we used normalized M_com_, which is defined as the M_com_ exerted at lift onset normalized by the sign of the target moment. A positive value of normalized M_com_ denotes compensatory moment exerted in the correct direction. We were primarily interested in two stages: *transfer learning*, and *post-transfer*. Specifically, the transfer learning consists of the four transfer trials (T1–T4, Figure 1B) which could be compared with the first four trials from the control group (C1–C4, Figure 1B). The post-transfer trials consist of the first trial from Block 2, 3, and 4. We used mixed-design ANOVAs for most of our analyses unless otherwise specified. The statistical factors are presented in the results.

Sphericity assumptions were tested for all analyses (Greenhouse–Geisser analysis) and the results were corrected when appropriate. All tests were performed at the *p*<0.05 significance level. Post hoc tests were performed with Bonferroni corrections.

## Results

### Learning compensatory moment with the right hand in the first block reached plateau after 3 trials

**Figure 2:**
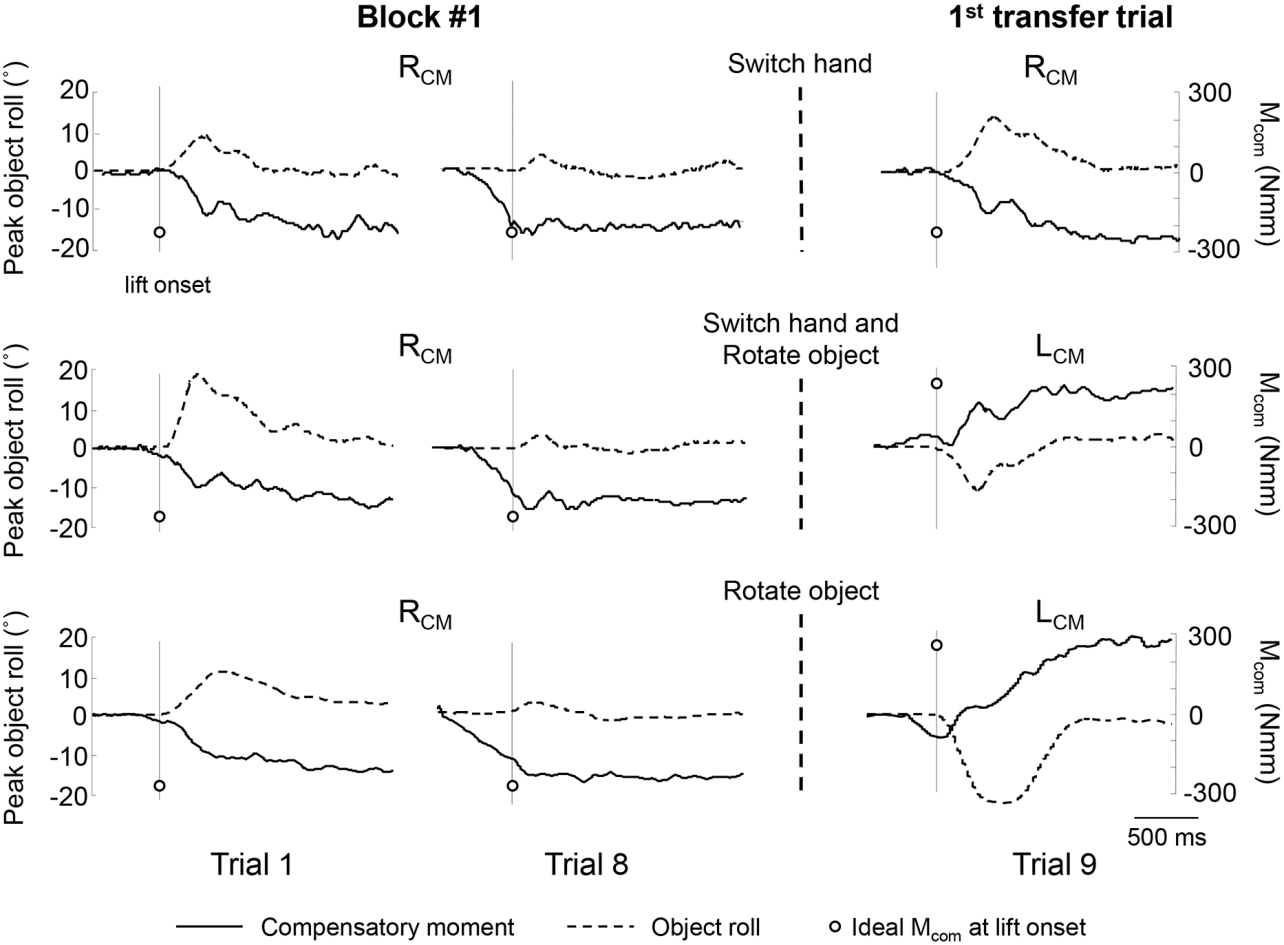
Compensatory moment and object roll. The figure shows the time course of compensatory moment (M_com_; solid line) and object roll (dashed line) on pre-transfer trials (Block 1, Trials 1 and 8) and the first transfer trial (Block 2, Trial 1) from 3 representative subjects (S6, switch hand group; S23, switch hand and object rotation group; S28, object rotation group). All subjects started with the right CM. The solid vertical line in each panel denotes object lift onset. Circles denote the subjects’ “ideal” M_com_ at object lift onset. The ideal M_com_ is the M_com_ that subjects should generate at object lift onset to neutralize the external moment generated by the mass added to the object. The left and right vertical axes refer to object roll and M_com_, respectively. Negative and positive values of object roll denote counterclockwise and clockwise roll relative to the vertical, respectively.

Figure 2 shows the time course of object roll and compensatory moment (M_com_) from representative individual trials from the three transfer groups. Consistent with our previous work (see Introduction), on Trial 1 of Block 1 of all three groups, M_com_ magnitude at lift onset was close to zero as subjects were unaware of the object CM location, and therefore the object rolled in the direction of the added mass. However, by trial 8 subjects learned to produce M_com_ whose magnitude was close to that required to counter the external moment (white circle), thus significantly reducing object roll relative to Trial 1.

**Figure 3:**
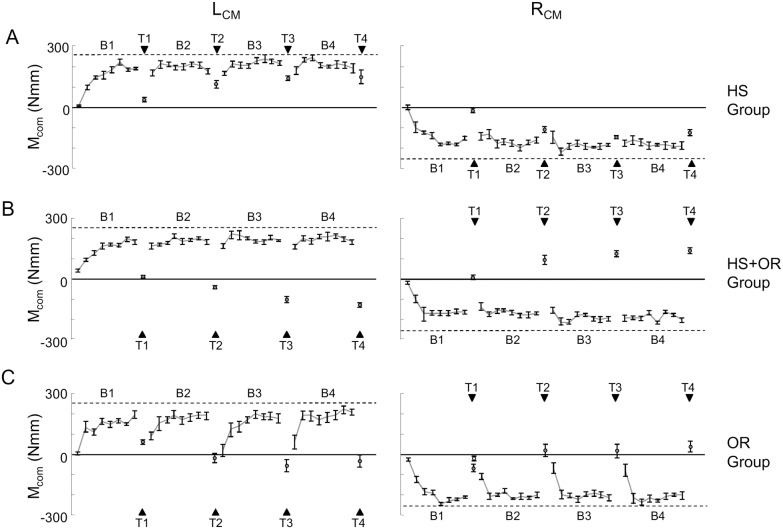
Compensatory moment across all trials. The relative compensatory moment (M_com_) is shown as a function of trial for each block of consecutive trials and transfer trials (T1 through T4). Data are separated for three groups and two blocked task CM conditions. Dashed horizontal lines denote the magnitude of M_com_ that the subjects should generate at object lift onset to neutralize the external moment during each block of consecutive trials. Black triangles denote M_com_ that subject should exert on the transfer trial. Data are averages of all subjects and vertical bars denote standard errors of the mean. The left and right columns represent L_CM_ and R_CM_ conditions, respectively. The top, middle, and bottom rows represent HS, HS+OR, and OR groups, respectively.

The patterns described for the three representative subjects shown in Figure 2 were found across all subjects. Figure 3 shows M_com_ averaged across all subjects for all transfer groups (separated plots for R_CM_ or L_CM_). Specifically, we found that all subjects learned to generate M_com_ required to minimize object roll within the first three trials of Block 1 (half-life of the exponential decay fits to M_com_: 1.29±0.16 trial for all subjects; no significant effect of CM or Group, 2-way ANOVA, *p*>0.05). Additionally, as expected from our previous work (Fu et al. 2010; Fu and Santello 2012), peak object roll decreased as a function of M_com_ (Pearson’s correlation coefficient, *r* = −0.72; *p*<0.001). Therefore, we will focus on M_com_ for the following analyses. In all transfer groups, on Trial 1 of Block 1 subjects produced very small M_com_ (normalized M_com_: 6.23±7.44 N·mm, 29.78±7.26 N·mm, and 12.01±8.97 N·mm, for HS, HS+OR, and OR respectively, averaged across CM conditions). Within the first 4 trials, all subjects learned to minimize peak object roll by generating appropriate moment at lift onset. On trial 4, subjects produced normalized M_com_ 148.38±15.16 N·mm, 161.88±12.64 N·mm, and 171.41±12.02 N·mm for HS, HS+OR, and OR respectively (averaged across CM conditions). 3-way ANOVA (Group×CM×Trial) revealed only a main effect of Trial (F_(3,90)_ = 68.23, *p*<0.001). Furthermore, no significant difference was found when using Trial 4–8 with 3-way ANOVA (Group×CM×Trial). Therefore, all groups learned the right-hand manipulation task in the first block similarly within the first 3 trials (Figure 3).

### Within-hand object rotation caused significant negative transfer at task-level

After object rotation, subjects in the OR group failed to generate the M_com_ with the magnitude and direction necessary to prevent roll (Figure 2C). This was confirmed by averaged group data. Subjects exerted normalized M_com_ of −59.6±14.9 Nmm averaged across CM conditions (Figure 3C). Furthermore, our trial sequence was designed such that all four transfer trials (T1–T4) could have been influenced by the preceding blocked task. This gives us a robust measure of transfer learning across multiple assessments. We found that all subjects gradually improved their performance as a function of repeated exposure to transfer trials (Figure 3C). Specifically, by the fourth transfer trial subjects exerted normalized M_com_ of 39.1±23.8 Nmm across CM conditions. Although the direction was correct, the magnitude of the M_com_ was still much less than the necessary one (255 Nmm), suggesting a negative transfer from the blocked trials. We compared the transfer learning trials from the OR group with a right-hand control group (3-way ANOVA; CM×Group×Trial) in which the block of consecutive trials with opposite CM caused by object rotation was replaced by breaks whose duration were equal to the time taken to perform eight consecutive trials (Figure 1B). This control group set the baseline behavior of learning object dynamics. As expected, learning of the manipulation task across the four transfer trials was much worse than learning across the four trials with breaks in between (significant effect of Group, F_(1,20)_ = 24.4, *P*<0.001), although both group improved over repeated (four times) exposure to the same CM conditions (main effect of Trial, F_(3,60)_ = 35.4, *P*<0.001).

### Left-hand learning is not affected in transfer trials regardless of object rotation

In contrast to the within-hand group (OR), the across-hand transfer groups (HS and HS+OR) did not exert M_com_ in the wrong direction on the first transfer trial (T1; Figure 2A and B). Instead, both groups exerted a M_com_ whose magnitude was close to zero as done on Trial 1 of Block 1 as if starting with no *a priori* knowledge of object mass location (normalized M_com_: 27.2±8.83 Nmm, −0.88±7.71 Nmm, HS and HS+OR groups, respectively, averaged across CM conditions; Figure 3A and B). The absence of the transfer continues as subjects gradually improved their performance as a function of repeated exposure to transfer trials similarly in the two across-hand transfer groups. Specifically, by the fourth transfer trial subjects exerted normalized M_com_ of 137.4±23.4 Nmm and 133.5±12.4 Nmm for HS and HS+OR groups, respectively. Additionally, we compared the transfer learning trials from the HS and HS+OR group with a left-hand control group in which blocked trials with right hand was replaced by breaks whose duration was equal to the time taken to perform the eight consecutive trials (Figure 1B). The right-hand blocked trials in the across-hand transfer groups did not influence the learning with the left hand regardless of object rotation, as indicated by the similarity of adaptation of M_com_. Three-way ANOVA (CM×Group×Trial) revealed only a significant main effect of Trial (F_(3,90)_ = 62.2, *P*<0.001), but not CM or Group.

### OR group exhibited significant ‘interference’ in compensatory moment on post transfer trials

**Figure 4:**
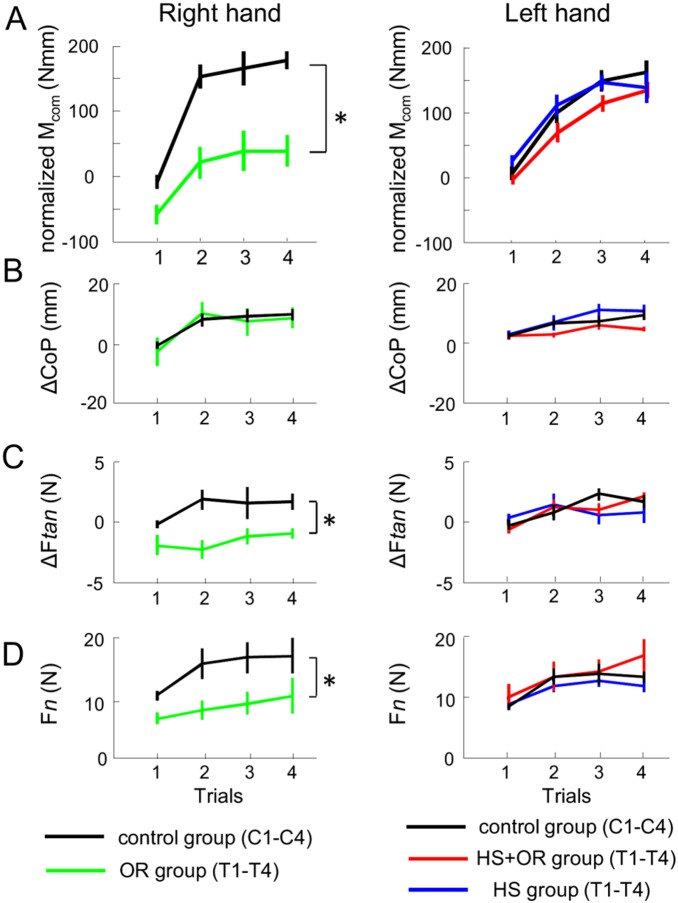
Task-related variables as a function of exposure to transfer trials. Data from the OR group (green) and right hand control (black) are shown on the left, whereas data from the HS group (blue), HS+OR groups (red), and left hand control (black) are shown on the right. Data from transfer trials (T1–T4) are from the transfer groups, whereas data from regular trials (C1–C4) are from the control groups. Panels A, B, C, and D show normalized compensatory moment (M_com_), digit placement (ΔCoP), digit load force sharing (ΔF*tan*), and grip force (F*n*), respectively. The asterisks denote significant differences (*p*<0.05). Data are averages of all subjects and vertical bars denote standard errors of the mean.

**Figure 5:**
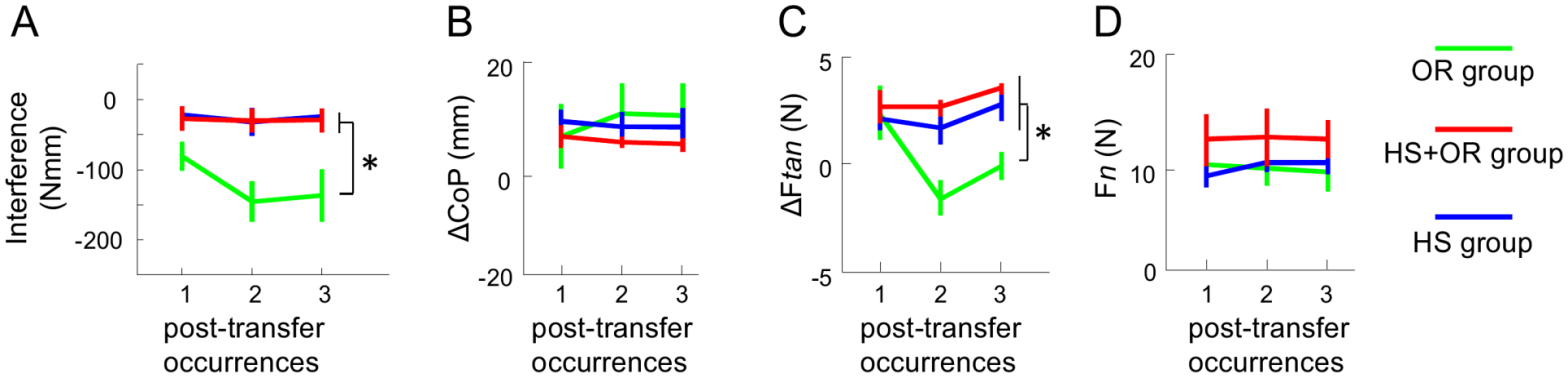
Interference in post-transfer trials. Panel A shows the *interference* (see text for details) across three post-transfer occurrences for three transfer groups, HS (blue), HS+OR (red), and OR (green). Panels B–D show digit placement (ΔCoP) and digit forces (ΔF*tan* and F*n*) from three transfer groups across three post-transfer trials. The asterisks denote significant differences (*p*<0.05) obtained from post hoc comparisons between groups across multiple occurrences (see text for details). Data are averages of all subjects and vertical bars denote standard errors of the mean.

As we asked subjects to resume the blocked task after each transfer trial, we could evaluate the ‘*interference*’ on the first trial of Block 2, 3, and 4 (Figure 4). The *interference* was calculated as the difference of normalized M_com_ between first post-transfer trials and the mean of the last five pre-transfer trials (e.g., Trial 1 Block 2 vs. Trial 4–8 Block 1). A negative value of this index would indicate that subjects performed worse in post-transfer trials than in pre-transfer trials. We found that subjects in OR exhibited large performance degradation in all the post-transfer trials that required subjects to re-adapt to perform the previously learned manipulation. In contrast, performance by HS and HS+OR groups after each transfer trial degraded to a smaller extent (−26.3±10.8 Nmm, −29.0±8.5 Nmm, and −121.4±14.9 Nmm for HS, HS+OR and OR, respectively, averaged across CM conditions and trials, Figure 5A). This was confirmed by an ANOVA showing a significant main effect of group (F_(2,30)_ = 11.1, *p*<0.001). Post hoc tests revealed that subjects in the OR group had significantly larger *interference* magnitudes relative to HS and HS+OR (*p*<0.05). In addition, the after-effect indices of HS and HS+OR groups were both significantly negative (one sample t-test, *p*<0.05).

### Inability to modulate digit forces during negative transfer

We compared the trial-by-trial modulation of digit level variables (i.e., digit positions and forces) between the transfer groups and the control groups. First, we examined the modulation of ΔCoP (i.e., relative digit positions). For the within-hand group (OR), we found that subjects modulated digit positions similarly to the subjects in the right-hand control group. Three-way ANOVA (CM×Group×Trial) revealed only a significant main effect of Trial (F_(3,60)_ = 8.2, *P* = 0.002), but not CM or Group. For the across-hand groups (HS and HS+OR), we also found that subjects modulated digit positions similarly to the subjects in the left-hand control group. Three-way ANOVA again revealed only a significant effect of Trial (F_(3,90)_ = 13.7, *P*<0.001), but not CM or Group (Figure 4B).

Second, we examined the modulation of ΔF*tan* (i.e., digit load force sharing). For the within-hand group (OR), subjects failed to modulate the ΔF*tan* across transfer trials to the same extent as the right-hand control. Three-way ANOVA revealed a significant main effect of Group (F_(1,20)_ = 14.5, *P* = 0.001). In contrast, no difference was found between the across-hand groups and the left-hand control group. Three-way ANOVA revealed only a significant main effect of Trial (F_(3,90)_ = 6.95, *P* = 0.001), but not CM or Group (Figure 4C).

Lastly, we examined the modulation of F*n* (i.e., digit grip forces). The within-hand group exerted less grip forces than the right-hand control. Three-way ANOVA revealed significant effect of Trial (F_(3,60)_ = 5.97, *P* = 0.005) and Group (F_(1,20)_ = 5.9, *P* = 0.024). In contrast, no difference was found between the across-hand groups and the left hand control group. Three-way ANOVA revealed only a significant effect of Trial (F_(3,90)_ = 21.8, *P*<0.001), but not CM or Group (Figure 4D).

In summary, our results suggest that subjects were not able to modulate digit load forces across all transfer trials for the OR group where negative transfer occurred, whereas the trial-to-trial digit position modulation was not affected. For the across-hand transfer groups, subjects behaved similarly as the control groups, indicating completely independent learning of the contralateral hand relative to the hand that learned the manipulation task across consecutive trials. Additionally, we also compared the digit-level variables across three transfer groups on post transfer trials (Figure 5B, C, and D). We found a significant main effect of CM on ΔCoP (three-way ANOVA, F_(1,30)_ = 10.6, *p* = 0.003), but no effect of Trial or Group. For F*n*, no significant effect was found. For ΔF*tan*, three-way ANOVA revealed a main effect of CM (F_(1,30)_ = 30.3, *p*<0.001) and a significant interaction Trial×Group (F_(3,90)_ = 3.11, *p* = 0.04). Post hoc analyses confirmed a significantly smaller ΔF*tan* in the within-hand transfer group OR than in the across-hand transfer groups (HS and HS+OR), and on the second and third post-transfer trial (*p*<0.05). These results indicate that the inability to modulate digit load forces correlates with the significant ‘*interference*’ found in the OR group.

## Discussion

The main finding of the present study is the different effect of object rotation versus switching hand on transfer of learned unconstrained manipulation. Specifically, we found a significant negative transfer of M_com_ in the within-hand OR condition (H2 supported), but zero transfer of M_com_ for both for the HS and HS+OR conditions across all four transfer trials (H1 unsupported). These findings extend previous work by showing that failure of transferring learned manipulation across hands is not due (1) to having experienced manipulation at constrained contacts, or (2) to a limited exposure and assessment on only one transfer trial. These results are discussed in the context of how dexterous manipulation is learned and represented, as well as previous work on across-arm transfer of reaching movements.

### Learning transfer of manipulation: digit placement and forces

First, we would like to point out that, unlike task-level M_com_, there is no ‘correct’ solution for digit forces and positions because subjects could have used an infinite number of combinations of digit-level variables and still attain a consistent manipulation performance by generating the same M_com_ [19]. It has been demonstrated that, once M_com_ is learned, the trial-to-trial modulation of digit positions and forces are not independent, suggesting active control of these two variables mediated by task-level goal [19], [20]. Therefore, unlike M_com_, we cannot define transfer at digit level as ‘positive’ or ‘negative’. However, it was also shown that when digit positions are not constrained, subjects tend to modulate the digit position in a way such that digit load force is more uniformly distributed between thumb and index finger [19]. Previous studies have shown that uniformly distributed load forces learned for lifting symmetrical object to match object weight could be transferred across hand. Therefore, it is theoretically possible that subjects could have benefited from implementing the digit position strategy learned in the blocked trials (e.g., thumb higher than index finger for CW torque) to the transfer trials. However, our data do not support this interpretation. In fact, all three transfer groups showed modulation of digit positions across four transfer trials similar to the corresponding control group (Figure 4B). This indicates that the learned digit position strategies obtained in the blocked trials did not affect subjects’ modulation of digit positions in transfer trials regardless of object rotation and hand switch. Furthermore, as for digit forces, subjects in OR group were not able to modulate their digit forces to the same extent as the right-hand control group, whereas performance by the across-hand transfer groups again were not different from that of the control groups. Overall, it appears that neither digit force or position adaptation in the right hand could affect manipulation performance with the left hand, thus resulting in completely independent learning of the left hand. In contrast, for the OR group, while digit position modulation remained unaffected by the blocked trials, the digit force modulation failed to produce the task torque. Previous studies have shown differences between sensorimotor mechanisms for the control of digit positions and forces [24], [25]: digit placement is primarily mediated by vision during reaching and prior to object contact, whereas digit forces are mediated by non-visual sensory feedback after contact. Furthermore, successful manipulation requires digit forces to be modulated to digit positions to ensure attainment of the desired M_com_ after making contact with the object. Based on this serial order of the execution of digit positions and forces, our data clearly indicate that digit position modulation in transfer trials was not affected by the preceding manipulation context. However, our data cannot provide direct evidence for failure of digit force control as the underlying cause of the failure of M_com_ transfer in the OR condition. Alternative interpretations are possible, as digit force control might not be affected by the preceding manipulation context, but subjects might still have modulated digit forces to generate an incorrect M_com_ due to a negative effect on task-level representations. In fact, the latter interpretation is more likely, since it has been shown that on the first trial after the hand was rotated (i.e., grasping from the back of the object), subjects were able to produce correct digit forces at constrained contacts, thus leading to positive transfer [12].

### Learning and transfer of manipulation: task representation

Bursztyn and Flanagan [12] did a series of transfer experiments using an inverted T-shaped object with constrained digit positions (collinear contacts). By comparing the peak object roll on the first trial of transfer block and the first trial of the first block, they showed negative transfer in a similar OR condition and zero transfer in a HS and HS+OR condition. The results of the present study are consistent with those from previous studies at the task level about the first trial transfer, while extending their findings to multiple transfer trials. The first result indicates that learned manipulation could induce interference to transfer contexts in which a context change occurs in both R_E_ and R_I_. Additionally, we have also demonstrated that such interference persisted even when visual geometric cue about object mass distribution was provided and no ‘object rotation’ was performed, as long as the learned context and transfer context have the opposite direction in both R_E_ and R_I_ [26]. In the current study, using our new trial sequence, we consistently showed that the interference (negative transfer) was found across multiple exposures to transfer trials for within-hand OR condition (Figure 4A). Furthermore, we also found that the transfer task itself, although being performed for one trial each time between blocks and not fully learned, could interfere with the subsequent recall of a learned manipulation in the post-transfer blocks (Figure 5A). Our results are also consistent with Bursztyn and Flanagan (2008) showing zero transfer on the first transfer trial, regardless of object rotation, when the hand is switched. Additionally, we also demonstrated zero transfer on the following transfer trials (Figure 4A). This contradicts our initial hypotheses that partial positive transfer and negative transfer could be found for HS and HS+OR groups, respectively, if multiple transfer trials were evaluated. Interestingly, this result would suggest that learning of manipulation tasks is quite different from learning of reaching tasks. However, we think that this difference could be explained by a general framework developed for reaching studies (see below).

### Comparison with reaching transfer studies

Besides dexterous manipulation tasks, there are many studies that have used reaching movements to investigate learning transfer. In these tasks, subjects usually have to adapt to uncommon dynamics (e.g., force fields, FF), or sensorimotor mapping (e.g., visuo-motor rotations). Comparison between transfer studies of tasks involving arm versus hand control using different motor tasks has to be taken with caution, since there are subtle differences between the two sensorimotor systems. For instance, perturbations delivered during reaching movements tend to be more complex and less familiar to the subjects, and thus take longer to adapt, whereas perturbations induced by changing object physical properties can be less challenging and take only a couple of trials to learn [27]. Nevertheless, reaching and manipulation also share common components, especially between FF tasks and our object lifting tasks, as they can both be considered as dynamic perturbations to point-to-point hand movements. We recently showed that within-hand transfer could be interfered by a previously learned manipulation in an ABA block design similar to the interference found in reaching studies [26]. Furthermore, our new multi-trial evaluation of transfer allows comparison of our results with how learning transfer is evaluated in reaching studies, although the assessment of learning rate is still not feasible for our manipulation task.

Using a rotating room to generate Coriolis force to reaching movement, DiZio and Lackner [28] tested subjects’ normal reaching with left or right hand as result of learning transfer following adaptation of right-hand pointing movement in a rotating room. It was found that left-hand reaching showed an after-effect in the form of small end-point position error, whereas the right hand showed an after-effect in the form of significantly curved reaching trajectory. The authors argued that the kinematic representation of the perturbation was more ‘central’, and therefore it could be transferred to the contralateral arm. Criscimagna-hemminger and colleagues [22] asked subjects to adapt to velocity-based curl fields with the dominant (right) arm or non-dominant (left) arm, and subsequently tested them with the contralateral arm in either the same or opposite force fields. It was shown that, when transferring the reaching movement from the dominant to the non-dominant arm, subjects had positive transfer with the same field and negative transfer with the opposite field. However, zero transfer was observed for non-dominant to dominant arm transfer groups. This result was interpreted as evidence that subject could transfer learned a force field in an extrinsic, but not intrinsic coordinate frame. However, a subsequent study demonstrated that such differential transfer can be observed only when the perturbation was introduced abruptly, but not gradually [29].

These seemingly disparate findings may be explained by a more generic model from Berniker and Kording [30]. They proposed that the sensorimotor system has two internal estimates for a given task: the property of the world and the property of the limb. Estimates of the world represent the knowledge that is independent of the motor apparatus, thus being similar to the notion of adaptation in R_E_. The estimates of the limb represent the knowledge of specific motor apparatus, thus being similar to the notion of adaptation in R_I_. Moreover, this framework also assumes that adaptation of the world parameter can be transferred across cerebral hemispheres, whereas the adaptation in the limb would be hemisphere specific. This framework utilizes Bayesian inference to assign the source performance error, i.e., which parameter estimate needs to be updated for adaptation. Essentially, this theoretical framework assumes that the sensorimotor system assign the source of error to these two estimates with a ratio α. The ratio between the world and limb was set to be 0.4 for right arm reaching in force field studies, i.e., limb parameters were updated more when an error occurs. Importantly, the value of α could be changed due to differences in motor task and training schedules. For instance, if the adaptation is performed by the left arm (non-dominant arm), the ratio was changed to 0.1 as uncertainty increases for the left arm because subjects are less familiar with its dynamics [31], which allows to explain zero transfer from left arm adaptation to right arm.

According to this theory, our finding could be interpreted as a low ratio for assigning error to world estimates and body estimates in right-hand blocked learning of a manipulation task similar to the left-arm reaching in force fields, thus driving the sensorimotor system to adapt mostly in the limb parameters, i.e., intrinsic coordinate frame. However, an intriguing question remains as why the ratios of adaptation in different coordinates frames are different between reaching tasks and object lifting tasks. A recent reaching study demonstrated that the different contextual cues influence the magnitude of within-hand generalization [32]. We speculate that the effectiveness of contextual cues may also cause the difference in across-hand learning transfer of different motor tasks, since strong context cues exist when a physical object is involved in manipulation tasks. Such cues are not present in most reaching tasks [26].

### Conclusions

The present study demonstrated that learned object manipulation is negatively transferred after object rotation, which was shown as impaired digit force control. Furthermore, learned manipulation cannot be transferred across hands, despite the fact that digit positions were not constrained and subjects were exposed to multiple transfer trials. This result suggests that, unlike reaching in force fields, object manipulation is learned in an end-effector (i.e., hand) specific fashion.
